# Cannabinoid CB_2_ Receptors Regulate Central Sensitization and Pain Responses Associated with Osteoarthritis of the Knee Joint

**DOI:** 10.1371/journal.pone.0080440

**Published:** 2013-11-25

**Authors:** James J. Burston, Devi Rani Sagar, Pin Shao, Mingfeng Bai, Emma King, Louis Brailsford, Jenna M. Turner, Gareth J. Hathway, Andrew J. Bennett, David A. Walsh, David A. Kendall, Aron Lichtman, Victoria Chapman

**Affiliations:** 1 Arthritis Research UK Pain Centre, University of Nottingham, Nottingham, United Kingdom; 2 School of Life Sciences, University of Nottingham, Nottingham, United Kingdom; 3 Department of Radiology, University of Pittsburgh, Pittsburgh, Pennsylvania, United States of America; 4 University of Pittsburgh Cancer Institute, Pittsburgh, Pennsylvania, United States of America; 5 Department of Pharmacology and Toxicology, Virginia Commonwealth University, Richmond, Virginia, United States of America; University of Arizona, United States of America

## Abstract

Osteoarthritis (OA) of the joint is a prevalent disease accompanied by chronic, debilitating pain. Recent clinical evidence has demonstrated that central sensitization contributes to OA pain. An improved understanding of how OA joint pathology impacts upon the central processing of pain is crucial for the identification of novel analgesic targets/new therapeutic strategies.

Inhibitory cannabinoid 2 (CB_2_) receptors attenuate peripheral immune cell function and modulate central neuro-immune responses in models of neurodegeneration. Systemic administration of the CB_2_ receptor agonist JWH133 attenuated OA-induced pain behaviour, and the changes in circulating pro- and anti-inflammatory cytokines exhibited in this model. Electrophysiological studies revealed that spinal administration of JWH133 inhibited noxious-evoked responses of spinal neurones in the model of OA pain, but not in control rats, indicating a novel spinal role of this target. We further demonstrate dynamic changes in spinal CB_2_ receptor mRNA and protein expression in an OA pain model. The expression of CB_2_ receptor protein by both neurones and microglia in the spinal cord was significantly increased in the model of OA. Hallmarks of central sensitization, significant spinal astrogliosis and increases in activity of metalloproteases MMP-2 and MMP-9 in the spinal cord were evident in the model of OA pain. Systemic administration of JWH133 attenuated these markers of central sensitization, providing a neurobiological basis for analgesic effects of the CB_2_ receptor in this model of OA pain. Analysis of human spinal cord revealed a negative correlation between spinal cord CB_2_ receptor mRNA and macroscopic knee chondropathy.

These data provide new clinically relevant evidence that joint damage and spinal CB_2_ receptor expression are correlated combined with converging pre-clinical evidence that activation of CB_2_ receptors inhibits central sensitization and its contribution to the manifestation of chronic OA pain. These findings suggest that targeting CB_2_ receptors may have therapeutic potential for treating OA pain.

## Introduction

Osteoarthritis (OA) is one of the most common causes of chronic pain with individuals experiencing pain at rest, on weight bearing [1], and pain from sites distal to the joint [2], [3]. The spread of pain to areas away from the diseased joint [2] suggests that changes in the central processing of sensory inputs contribute to OA pain. Indeed a recent study provided psychophysical and imaging evidence supporting a contribution of central sensitization to OA pain [4]. Central sensitization plays a pivotal role in the switch from acute to chronic pain mechanisms [5], [6] and the manifestation of altered sensory responses, such as touch-evoked pain (mechanical allodynia), in models of chronic pain [7]. Spinal neuronal facilitation and the activation of spinal microglia and astrocytes [8], [9], [10] play fundamental roles in these processes. Experimental models of OA, such as the intra-articular injection of monosodium acetate (MIA), are associated with joint pathology [11], [12] and pain behaviour [13], [14], [15], [16], [17] comparable to clinical OA. We have demonstrated the facilitation of spinal neuronal responses [17], and the activation of spinal microglia and astrocytes [18], [19], [20], [21] has also been demonstrated, in the MIA model of OA pain. These observations support the use of this model of OA pain to study the neurobiological mechanisms underpinning the manifestation of central sensitization associated with OA.

Current analgesic treatments for OA pain have either incomplete efficacy, or potentially severe adverse events [22], limiting treatment options for OA sufferers. The discovery of a contribution of central sensitization to OA pain supports the investigation of novel drug targets within the central nervous system for the treatment of OA pain. The analgesic effects produced by activation of the cannabinoid (CB) receptor system are well documented and mediated by multiple sites of action [23]. Dynamic changes in the spinal endocannabinoid system are evident in the MIA model of OA pain; endocannabinoid levels are elevated in the spinal cord and tonically regulate neuronal activity via CB_1_ and CB_2_ receptors [17]. Over-expression of CB_2_ receptors significantly attenuated mechanical allodynia in a mouse model of OA pain, without influencing joint pathology, suggesting that CB_2_ receptors can regulate OA pain responses via sites distinct from the joint [24]. It is well accepted that activation of spinal CB_2_ receptors attenuates pain behaviour in models of neuropathic pain [25], [26], via modulation of microglia and astrocytic pro- and anti-inflammatory responses [27], [28]. We hypothesised that activation of CB_2_ receptors would attenuate OA pain responses in a model of OA pain, and that these effects would be associated with a decrease in systemic and spinal markers of central sensitization.

The aim of this study was to determine whether activation of the CB_2_ receptor attenuates pain behaviour in the MIA model of OA pain, and then to investigate the sites of action, and mechanisms by which, analgesic effects were produced. The contribution of a spinal site of action was evaluated by electrophysiological recordings of spinal neurones, quantification of spinal CB_2_ receptor gene expression and immunohistochemical quantification of the cellular location of CB_2_ receptors in the spinal cord in MIA-treated rats, compared to controls. The influence of the CB_2_ receptor agonist JWH133 on spinal markers of central sensitization was determined, providing a mechanistic basis for the analgesic effects of this intervention in this model. The final series of experiments determined whether spinal CB_2_ receptor expression is correlated with knee joint damage (macroscopic chondropathy score) in human post mortem samples.

## Methods

Studies were in accordance with UK Home Office Animals (Scientific Procedures) Act (1986) and the International Association for the Study of Pain guidelines and were approved by ethical review board at the University of Nottingham. Data are presented in line with the ARRIVE guidelines. Male Sprague Dawley rats (arrival weight of 130–150 g, Charles River U.K.) were used. CB_2_ homozygous knockout mice (CB_2_ KO, 005786, B6.129P2-Cnr2tm1Dgen/J, n = 9) and wild type mice (000664, C57BL/J6, n = 5) were provided by Professor Lichtman (File S1). All animal studies were conducted in a manner that minimised animal distress, and euthanization of the animal occurred via an appropriate S1 technique (as listed by the UK Home Office), or by terminal overdose with sodium pentobarbital followed by transcardial perfusion (once animals were areflexic).

### MIA model induction

Adult male Sprague Dawley rats (180–200 g at time of induction) were anesthetised with isoflurane (2.5–3%) in 100% oxygen (1 L per min) and received a single intra-articular injection of monosodium iodoacetate (MIA; 1 mg/50 µl; Sigma U.K.) in saline through the infra-patellar ligament of the left knee [17]. Control rats received intra-articular injection of 50 µl of saline. Pain behaviour was quantified as a change in hindlimb weight-distribution (Incapacitance Tester, Linton Instrumentation, U.K.) and hindpaw mechanical withdrawal thresholds (von Frey testing), as previously described [17].

### Systemic drug administration and pain behaviour

MIA-treated rats received daily subcutaneous injection of either JWH133 (a CB_2_ receptor agonist with a Ki of 3.4 nm and approximately 200 fold selectivity for CB_2_ over CB_1_ receptors [29]) at a dose of 1 mg/kg at a volume of 1 ml/kg (n = 8), or vehicle, 5% ethanol. 5% emulphor and 90% saline (n = 8) from day 0–28 post-MIA injection. Changes in hind limb weight-distribution and hindpaw mechanical withdrawal thresholds were measured in a blinded fashion.

### ELISA assays

Microwell strips for Interleukin-1 beta (IL-1β), tumor necrosis factor-alpha (TNF-α), and Interleukin 10 (IL-10) were washed with wash buffer. Standard curve solution was added to standard curve wells, 50 µl of sample serum + 50 µl sample diluent and 50 µl of biotin conjugate were mixed together (mixture A) and added to the wells and incubated for 2 hours at room temperature with gentle agitation. Mixture A was removed and wells washed and 100 µl of diluted Streptavidin-HRP (mixture B) was added and incubated for 1 hour at room temperature with gentle agitation. Mixture B was removed and wells washed and 100 µl of TMB substrate solution was added to the wells and incubated for 20 min at room temperature with gentle agitation until the highest standard curve point had developed (dark blue colour). Enzymatic reaction was stopped by the addition of 100 µl of stop solution. Plates were read on a spectrophotometer using 405 nm reference wavelength, and cytokine concentration determined from the standard curve. All samples and standards were run in triplicate.

### In vivo electrophysiology

Rats were anaesthetised, placed in a stereotaxic frame and a laminectomy (L4-5) was performed [17]. Extracellular single-unit recordings of deep wide dynamic range dorsal horn neurones were made, action potentials were digitised and quantified with a CED micro1401 interface and Spike 2 software (Cambridge Electronic Design, UK). Von Frey monofilaments (bending forces 10, 15, 26 and 60 g) were applied to the plantar surface of the neuronal receptive field for 10 seconds and mean frequency of firing recorded.

Effects of a spinal administration of the CB_2_ receptor selective agonist [29] JWH-133 (8-486 ng/50 µl based on [25] n = 6 neurones in 6 rats for each group) or vehicle (0.0005–0.03% EtOH in distilled water; n = 7 neurones in 7 MIA-treated rats and n = 9 neurones in 9 saline-treated rats, respectively) on mechanically-evoked responses of dorsal horn neurones were studied. Dorsal horn neurones were located between 700–900 µm from the surface of the spinal cord and were located in laminae V–VI.

### RNA extraction and cDNA synthesis

50 mg of frozen rat spinal cord tissue was homogenized in 2 ml of ice cold Tri reagent (Sigma-Aldrich, UK) and RNA purified as previously described [30]. mRNA was isolated from total RNA using Dynabeads mRNA purification kits (Life Technologies). For cDNA synthesis, mRNA was reverse transcribed using Superscript III reverse transcriptase (Life Technologies). RNA was extracted from human spinal cord lumbar segment L4 (that had been collected 24–48 hours post mortem and quick frozen in melting isopentane) using a Pure Link™ FFPE RNA Isolation Kit (Life Technologies). 200 ng of total RNA was then reverse transcribed as above.

### Taqman quantitative real time polymerase chain reaction

Gene expression was quantified by Taqman quantitative real time PCR using the relative standard curve method [31]. Beta-actin was used as an invariant reference gene for normalization of expression between samples. Primers and probes were designed using Primer Express3 software (Applied Biosystems, UK), and synthesised by MWG Biotech (Germany), see File S1. CB_2_ receptor gene expression was measured in ipsilateral and contralateral rat lumbar (L3–L5) spinal cord. Gene expression levels of CB_1_, CB_2_, GFAP and TRPV1 receptor and the enzyme COX2 were quantified in human lumbar (L3–L5) spinal cord tissue (see File S1).

### Immunohistochemistry

Rats were overdosed with sodium pentobarbital and transcardially perfused with saline and 4% paraformaldehyde (Sigma, U.K). The lumbar spinal cord was removed, post-fixed and stored in 30% sucrose. Immunohistochemistry (following citrate buffer antigen retrieval) for spinal cord sections (40 µm) used goat anti Iba-1 (1∶500, Abcam, Cambridge, UK), rabbit anti-CB_2_ (ab3561 1∶300, Abcam, Cambridge, UK), mouse anti-GFAP (1∶100, Fisher scientific UK), mouse anti-Neu-N (1∶100, Anti-NeuN, clone A60 Millipore, Germany) antibodies. Secondary antibodies were Alexafluor 568 conjugated Donkey anti-goat (1∶200), Alexafluor 568 conjugated Donkey anti-mouse secondary antibody and Alexafluor 488 conjugated Donkey anti-rabbit (1∶200) (Molecular probes, Oregon). Images were captured and processed identically and any contrast enhancement applied consistently for each image, as described in [19]. All images were digitally captured with an 8 bit camera, thus giving grey level (intensity) values of 0–255.

### NIR660-Mbc94 CB2 receptor probe assay

The details of the synthesis of the CB2 receptor probe NIR6660-Mbc84 are included in File S1. Lumbar spinal cord sections (10 µm) on silane-prepared slides (Sigma Aldrich) were incubated in buffer A (50 mM Tris–HCl, 3 mM MgCl2, 0.2 mM EGTA, 100 mM NaCl) for 1 hour, and then buffer A+0.5% BSA (Buffer B) for 1 hour. Sections were incubated for 3 hours with either ethanol (0.1%), JWH133 (3 µM) or SR144528 (1 µM) made up in buffer B. Sections were incubated for 5 hours with NIR660-Mbc94 (3 µM) alone or in the presence of JWH133 (3 µM) or SR144528 (1 µM) in buffer B. Sections were washed, dried and mounted in Polyvinyl alcohol mounting medium with NPG, antifading (Sigma catalogue number: 10979). NIR660-Mbc94 staining was visualised and analysed using a Leica DMIRE2 fluorescence microscope, and Volocity 5.5 (PerkinElmer). A 300 ms image capture was used to ensure acquisition was in the linear dynamic range of the camera. Details of the Cresyl violet, Hoechst 33342, NIR660-Mbc94 staining protocol and Confocal Microscopy image acquisition are in File S1.

### Gelatin zymography

Rat spinal cords were homogenized in lysis buffer (50 mM Tris-HCl,, 150 mM NaCl, 1% Nonidet P-40, 0.1% SDS, 0.1% deoxycholic acid, 2 µg/ml leupeptin, 2 µg/ml aprotinin, and 1 mM PMSF PH. 7.4) and mixed for 3 h at 4°C. The homogenate was centrifuged at 15,000×g for 15 min at 4°C. Supernatant layer was removed and 20–30 µg of supernatant was diluted in Zymogram Sample Buffer (Bio-Rad Laboratories, Inc, 161–0764) and loaded onto precast Zymogram gels (10%, gelatin, 10-well, 30 µl, 8.6×6.8 cm (W×L), Bio-Rad Laboratories, Inc, 161–1113). Electrophoresis was performed in Tris-glycine buffer at 120–130 volts for 3 h. Gels were incubated for 3 hr at room temperature in 100 ml of 3% Triton X-100 on a rotary shaker and then with 200 ml of development buffer (Bio-Rad Laboratories, Inc 161–0766) for a further 1 hour, gels were then incubated in fresh development buffer at 37°C for 24–30 hr on a rotary shaker. Bands were visualised by Coomassie Blue staining. Purified MMP-9 and MMP-2 (5427-MM-010 and 924-MP-010, respectively, R & D systems) were used as positive controls. Images of gel zymograms were captured using a Li-Cor® ODYSSEY imaging system, using the gel scan function (linear manual setting of 6, contrast 50/100 and brightness 50/100). For densitometry analysis, we used IMAGE J (NIH open software with Macbiophotonics plugins) software.

### Chondropathy scoring of clinical samples

Post-mortem knee joints were obtained from recently deceased patients. The presence and nature of knee pain in these cases is not known. The articular cartilage integrity of the medial and lateral femoral condyles and tibial plateaux of the knee joints were determined by a single assessor as previously described [32], [33]. Chondropathy was graded 0 (normal) to 4 (subchondral bone exposure) and chondropathy scores were calculated as previously described [32], [33]. Total scores ranged from 0 to 400 for the joint, and left and right knee scores were summed.

### Statistical analysis

All statistics were calculated using Prism 5.0 software (Graphpad, La Jolla, USA). Data were analysed with either a one way or two way ANOVA, a t-test, or Spearman correlation (*p<0.05, **p<0.01, ***p<0.001). For data that did not pass normality testing non-parametric statistics were used. Analysis of ELISA data (TNFα and IL-1β) used a one sample t-test using detection limit of kit as hypothetical value as there was no variation in saline + vehicle group and MIA + JWH133 group, for IL-10 data a one-way ANOVA was used. Correlations between human gene expression and chondropathy or age were determined with either a Pearson correlation or Spearman correlation depending on whether data passed normality testing.

## Results

### CB_2_ receptor activation attenuates OA pain behaviour and spinal neuronal responses

**Figure 1:**
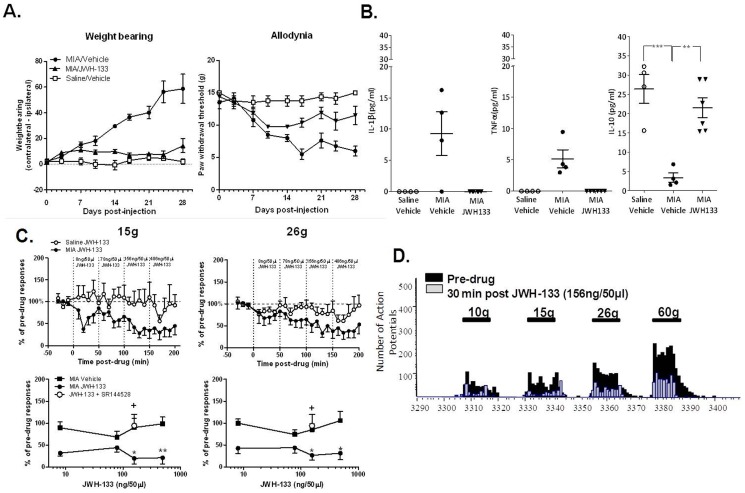
(A) Repeated systemic administration of the CB_2_ receptor agonist JWH-133 (1 mg/kg; day 1–28) attenuated MIA-induced changes in weight bearing (p<0.001 area under the curve analysis of MIA + Vehicle vs. MIA + JWH133) and mechanical withdrawal thresholds (p<0.001 area under the curve analysis of MIA + Vehicle vs. MIA + JWH133) of the ipsilateral hindpaw, n = 8 rats per group). (B) Effects of repeated systemic administration of the CB_2_ receptor agonist JWH-133 (1 mg/kg; day 1–28) on MIA-induced changes in serum levels of IL-1β, TNF-α and IL-10. Analysis of IL-1β and TNF-α used a one sample t-test using detection limit of kit as hypothetical value as there was no variation in saline + vehicle group and MIA + JWH133 group, for IL-10 data a one-way ANOVA and a Bonferroni post-hoc test was used, **p<0.01, ***p<0.001 (n = 4–6 rat serum samples per group). (C–D) Effects of spinal JWH-133 on noxious (15 g and 26 g) mechanically evoked responses of WDR neurones in MIA-treated rats (n = 6 neurones in 6 rats) and saline-treated rats (n = 6 neurones). Spinal administration of vehicle did not alter evoked responses of neurons in MIA-treated rats (n = 7 neurones in 7 rats). Effects of JWH-133 (156 ng/50 µL) were abolished in the presence of SR144528 (n = 6 neurones in 6 rats; 0.001 µg/50 µl) as indicated by open circle data point on bottom two panels. Data are expressed as mean maximal inhibition (% of pre-drug response) ± SEM. Statistical analyses were performed using a Kruskal Wallis test or Mann Whitney test as appropriate (*p<0.05, **p<0.01 for MIA-JWH133 versus MIA-Vehicle and + p<0.05 for MIA-JWH133 versus MIA-JWH+SR144528). (D) Representative trace of innocuous (10 g) and noxious (15–60 g) mechanically evoked responses of a single dorsal horn neurone before and 30 minutes following spinal administration of JWH-133 (156 ng/50 µL) in MIA-treated rats.

The ability of a selective CB_2_ agonist JWH133 to modulate OA pain behaviour and noxious-evoked responses of spinal neurones was investigated. As previously described, intra-articular injection of MIA into the knee joint of rats resulted in significant decreases in weight bearing on the ipsilateral hind limb and mechanical withdrawal thresholds of the ipsilateral hindpaw, compared to saline-treated rats (Figure SF1 in File S1). In line with our previous study [19], pain behaviour was significantly different to saline controls by day 7 post MIA injection, and was maintained for at least 28 days (Figure SF1 in File S1). Repeated systemic dosing with JWH133 significantly attenuated the development of pain behaviour (significant change in weight bearing from day 14 and mechanical withdrawal thresholds from day 10) in MIA-treated rats (Fig 1A). OA is associated with dynamic changes in levels of circulating cytokines (see references in [34], [35]), in the MIA model there was a trend towards increased serum levels of pro-inflammatory IL-1β and TNFα in MIA-treated rats, compared to saline-treated rats (Fig 1B). Levels of the anti-inflammatory cytokine IL-10 were significantly decreased in MIA-treated rats, compared to saline-treated rats (Fig 1B). Systemic JWH133 prevented the MIA-induced alterations in serum levels of cytokines (Fig 1B).

Given that the spinal cord plays a pivotal role in the integration and modulation of central sensitization, the potential contribution of a spinal site of action to the effects of the CB_2_ receptor ligand was investigated. Spinal administration of JWH133 in MIA-treated rats with established pain behaviour, significantly decreased innocuous and noxious mechanically (15 and 26 g) evoked firing of wide dynamic range (WDR) neurones, compared to the effect of vehicle in MIA-treated rats (Fig 1C, D). The inhibitory effects of JWH133 on evoked neuronal responses were dose-related and blocked by the CB_2_ receptor antagonist SR144528 (Fig 1C). Interestingly the inhibitory effects of JWH133 on evoked responses of spinal neurones were only observed in the model of OA pain and not control rats (Fig 1C), indicating a novel effect of this intervention in the model of OA.

### Increased expression of spinal CB_2_ receptors in the MIA model of OA pain

**Figure 2:**
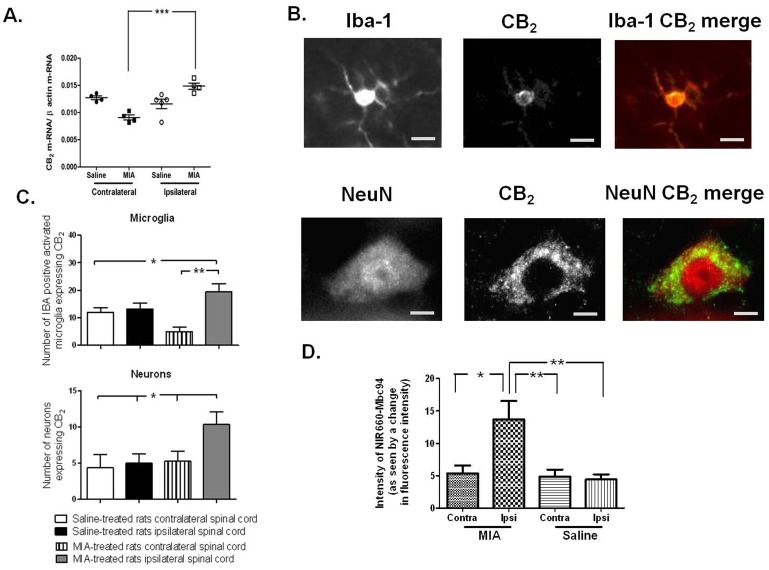
(A) CB_2_ mRNA expression in the ipsilateral and contralateral lumbar spinal cord of MIA- and saline-treated rats. Data are normalised to levels of β-actin (mean ± SEM, n = 4–5 per group), region/treatment comparison was performed with a one way ANOVA and Neuman-Keuls post-hoc test, ***p<0.001. (B) Representative images of CB_2_ receptor immunofluorescence localised with markers for neurones (Neu-N) and microglia (Iba-1) in the dorsal horn of spinal cord (scale bar  = 10 µm). (C) Quantification of the number of Iba-1 positive and Neu-N positive cells in the dorsal horn of the spinal cord which co-express CB_2_ receptor immunofluorescence. Data are expressed as mean ± SEM, statistical analysis were performed using a one way ANOVA followed by Bonferroni post-hoc (n = 5–6 sections per rat, 5–6 rats per group),*p<0.05, **p<0.01. (D) Quantification of CB_2_ probe (AU) in ipsilateral and contralateral dorsal horn quadrants in sections from MIA-treated (n = 5) and saline-treated (n = 4) rats (mean + SEM, 5–6 spinal cord sections per rat),*p<0.05, **p<0.01.

To investigate why there was a novel inhibitory effect of the CB_2_ receptor agonist in MIA-treated rats, the expression and localisation of CB_2_ receptors in the spinal cord was quantified in MIA-treated rats. At day 28 post model induction, CB_2_ mRNA levels were significantly increased in the ipsilateral spinal cord of MIA-treated rats, compared to the contralateral spinal cord, but there were no differences between MIA- and saline-treated rats (Fig 2A). Levels of CB_2_ mRNA in the ipsilateral and contralateral spinal cord of saline-treated rats were comparable. Immunofluorescence studies localised CB_2_ receptor protein in the dorsal horn of the spinal cord in the rat (Figure SF2A in File S1). There was a significant increase in the number of CB_2_ expressing Iba-1 positive activated (as indicated by an amoeboid morphology [19]) microglia (Fig 2B, C) and Neu-N positive neurones (Fig 2B, C), in MIA-treated rats, compared to the contralateral side, and compared to saline-treated rats. The number of GFAP positive cells (a marker of reactive gliosis) expressing CB_2_ receptor protein was negligible, indicating little expression of CB_2_ receptor protein by astrocytes in the spinal cord (data not shown). Validation experiments with the CB_2_ receptor antibody using spinal cord tissue from wildtype and CB_2_ receptor knockout mice were undertaken (Figure SF2B, C and SF3 in File S1). The number of CB_2_ positive Iba-1 positive microglia was lower in spinal cord from CB_2_ knockout mice (3±1 Iba-1 positive microglia per section), compared to wild type spinal cord (15±3 Iba-1/CB_2_ positive microglia per section) (5–8 sections per mouse, 3 mice per genotype. Similarly, numbers of CB_2_ positive Neu-N positive cells were lower in spinal cord from CB_2_ knockout mice (1±1 CB_2_ positive neurons per section), compared to wild type spinal cord (7±1 CB_2_ positive neurons per section).

Since some residual CB_2_ receptor immunofluorescence was apparent in the knockout tissue, a fluorescently labelled CB_2_ receptor antagonist NIR660-Mbc94 (Figure SF4-SF6 in File S1), was used to validate the observation that CB_2_ receptor expression is increased in the spinal cord in the model of OA pain. In order to establish that this fluorescent probe bound to CB_2_ receptors, a combination of CB_2_ selective ligand competition binding and confocal microscopy experiments were conducted (Figure SF7 in File S1). We report competition specific binding of NIR660-Mbc94 to CB_2_ receptors, and that the cellular binding pattern of NIR660-Mbc94 (intense staining of the surface of cells) is consistent with a plasma membrane localisation. CB_2_-selective NIR660-Mbc94 binding in the ipsilateral lumbar spinal cord of MIA-treated rats was a significantly increased compared to binding in the contralateral spinal cord of MIA-treated rats, and compared to the ipsilateral spinal cord of saline-treated rats (Fig 2D). These data support our immunohistochemical evidence that CB_2_ receptors are up-regulated in the spinal cord in this model of OA pain.

### Activation of CB_2_ receptors attenuates spinal mechanisms of central sensitization in the model of OA pain

**Figure 3:**
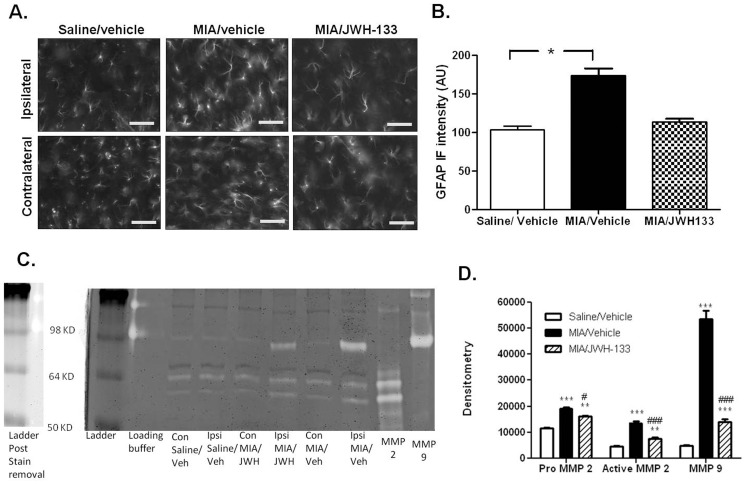
(A) Representative images of GFAP immunofluorescence, a marker of reactive gliosis, in the ipsilateral and contralateral dorsal horn of the spinal cord. Images are from rats that received intra-articular injection of saline or MIA, and treatment with vehicle or the CB_2_ receptor agonist JWH133 (1 mg/kg, days 1–28) (scale bar = 5 µm). (B) Quantification of ipsilateral GFAP immunofluorescence expressed as a % of the immunofluorescence for the contralateral spinal cord for the three treatment groups (n = 6–7 sections per rat, n = 4 rats per treatment). Systemic administration of JWH-133 significantly attenuated increases in GFAP immunofluorescence, compared to the effects of vehicle, statistical analysis was conducted via a one-Way ANOVA with a Bonferroni post-hoc test,*p<0.05. (C) Example gel zymography of MMP-9 and MMP-2 activity in the spinal cord from the various treatment conditions, as well as positive controls for purified MMP 2 and MMP 9. (D) Quantification of MMP-9 and pro and active MMP-2 activity in the ipsilateral spinal cord in MIA-treated rats and saline-treated rats. Systemic administration of JWH-133 significantly attenuated increases in MMP-9, MMP-2 and active MMP-2 activity in the spinal cord in MIA-treated rats, compared to the effects of vehicle. Data are expressed as mean densitometry ± SEM (n = 4 rats per group), statistical analysis one-way ANOVA and Bonferroni post-hoc test,**p<0.01, ***p<0.001 vs. saline/Vehicle; #p<0.05, ###p<0.001 vs. MIA/Vehicle.

The next series of experiments investigated why CB_2_ receptor activation inhibits spinal nociceptive processing in the model of OA pain, but not the physiological processing of noxious inputs in control rats. It is established that reactive gliosis plays a crucial role in the maintenance of central sensitization in chronic pain states [27], and activation of metalloprotease-2 and metalloprotease-9 (MMP-2 and MMP-9) has recently been implicated in the activation of astrocytes [36]. We postulated that the up-regulation of CB_2_ receptors in the spinal cord attenuates MMP-2 and MMP-9 activity and concomitantly the activation of astrocytes in the spinal cord, which puts a brake on the processes underpinning central sensitization. Consistent with our previous work [19], GFAP immunofluorescence, a marker of reactive gliosis, was significantly increased in the ipsilateral spinal cord of MIA-treated rats, compared to saline-treated rats (Fig 3A, B). In addition, we demonstrate for the first time that the pro- and active forms of MMP-2 are significantly elevated in the spinal cord of MIA-treated rats, compared to saline-treated rats (Fig 3C, D, Figure SF8A, B in File S1). A single band corresponding to MMP-9 activity was detected in the spinal cords of MIA-treated rats, (Fig 3C). Systemic administration of the CB_2_ receptor agonist JWH133 prevented the MIA-induced increase in spinal GFAP immunofluorescence (Fig 3A, B), and MMP-2 activity and MMP-9 activity in the spinal cord (Fig 3C, D). These data strongly support the hypothesis that activation of CB_2_ receptors inhibits essential cellular mechanisms associated with the manifestation of central sensitization in this model of OA pain.

### Are there changes in CB_2_ mRNA in human spinal cord associated with joint pathology

**Figure 4:**
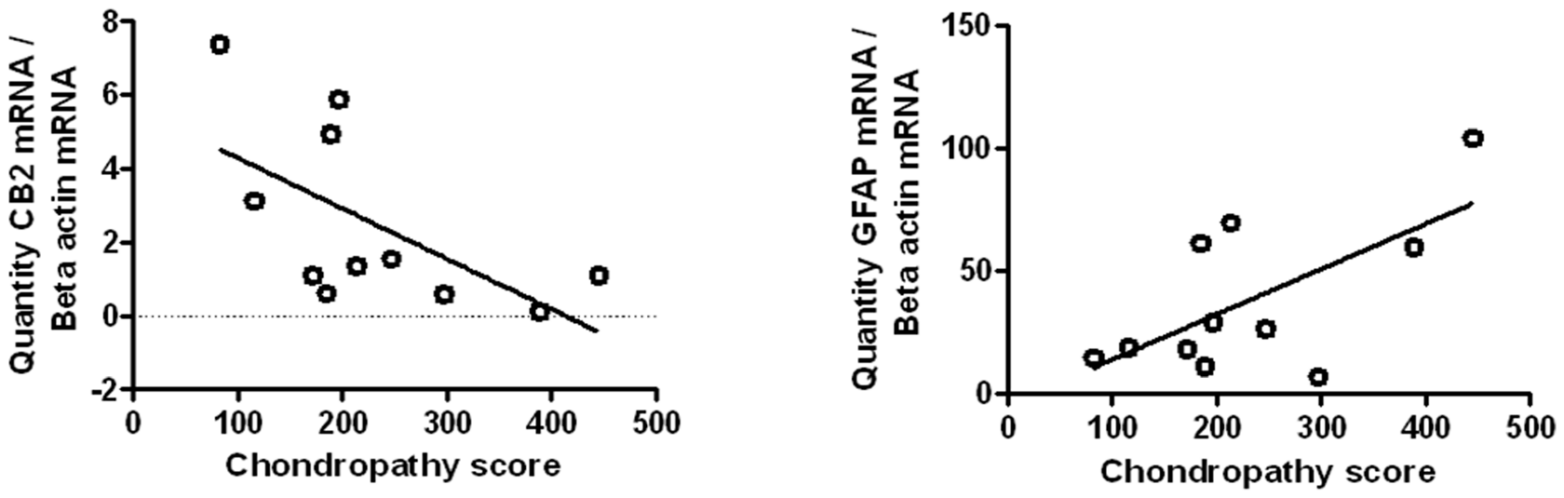
(A) CB_2_ receptor mRNA expression in the lumbar spinal cord was negatively correlated with macroscopic knee chondropathy scores. (B) GFAP mRNA expression in the lumbar spinal cord was positively correlated with macroscopic knee chondropathy scores. Data are expressed as mean (normalised to beta actin) ± SEM, statistical analysis (n = 11 separate spinal cord cases). Data were analysed with either a Pearson correlation or Spearman correlation depending on whether data passed normality testing.

Given that our data suggest that CB_2_ receptors attenuate central sensitization mechanisms and pain behaviour in the MIA model of OA pain, the final experiments investigated potential associations between knee joint damage (macroscopic chondropathy score) and CB_2_ receptor gene expression in human lumbar spinal cord (n = 11 cases). In addition, associations between spinal expression of GFAP, TRPV1 and COX2 mRNA and macroscopic chondropathy score were also determined in these cases. Macroscopic chondropathy score were within the range of: 83–523, unfortunately pain scores for these cases were not available. There was a significant negative correlation between joint chondropathy score and spinal CB_2_ mRNA expression (normalised to β-actin) in these samples (Fig 4A). There was no significant correlation between CB_2_ mRNA expression and age (Spearmans r: 0.23 p = 0.50). Spinal GFAP mRNA expression was positively correlated with chondropathy score (Fig 4B) and with age (Pearsons r value of 0.62 p = 0.04). There was no correlation between chondropathy score and spinal CB_1_ mRNA expression (Spearmans r: 0.38 p = 0.12), nor between chondropathy score and spinal COX-2 mRNA expression (Spearmans r: 0.20 p = 0.28), nor between chondropathy score and spinal TRPV1 expression (Pearsons r value of 0.42 p = 0.1831).

## Discussion

We have shown that the CB_2_ receptor agonist JWH133, which lacks centrally mediated side-effects (catalepsy and motor disturbances) seen with CB_1_ receptor agonists such as Δ^9^-THC [37], attenuated pain behaviour and spinal neuronal responses in a model of OA pain. The up-regulation of the inhibitory CB_2_ receptor system in the spinal cord in the model of OA pain, and its ability to modulate the cellular mechanisms underpinning the manifestation of central sensitization, suggest novel therapeutic potential of this target.

The experimental model of OA-like pain used in the present study produces clinically relevant joint pathology, distal allodynia and, by day 28, spinal correlates of central sensitization [17], thus mimicking clinical features of pain reported in OA patients [2]. Here we demonstrate that systemic administration of the CB_2_ receptor agonist (JWH133) attenuated the development and maintenance of pain OA behaviour. It is noteworthy that unlike CB_1_ receptor agonists [38], we did not observe any evidence of tolerance to the effects of repeated systemic administration of the CB_2_ receptor agonist over the course of the study. Activation of CB_2_ receptors has well described anti-inflammatory effects, attenuating pro-inflammatory signalling pathways mediated by interferon gamma [28], [39] and interleukin 1 beta [28] and CB_2_ receptor activation potentiates anti-inflammatory signalling mediated by interleukin-10 in other models of chronic pain [28]. Clinical OA is associated with changes in levels of cytokines [34], [35], [40] which can modulate pain through a variety of mechanisms including peripheral and spinal sensitization [28], [41], [42]. Here we demonstrate that circulating levels of pro- and anti-inflammatory cytokines are altered in the MIA model and that treatment with the CB_2_ receptor agonist prevented these changes in circulating cytokines.

The demonstration herein that a CB_2_ receptor agonist (JWH133) can attenuate pain behaviour and evoked spinal neuronal responses in MIA-treated rats which had established chronic pain behaviour, but not in control rats, is consistent with previous reports of a functional role of spinal CB_2_ receptors in modulating neuropathic pain responses [25], [28], [39], [43] and the observation that over-expression of CB_2_ receptors is associated with a reduced pain phenotype in MIA-treated mice [24]. The inhibitory effects of the CB_2_ receptor agonist in MIA-treated rats on spinal neuronal responses were blocked by the selective CB_2_ receptor antagonist SR144528. We report subtle, but significant, changes in the spinal CB_2_ mRNA in this model of OA pain. Levels of CB_2_ mRNA were significantly increased in the ipsilateral spinal cord of MIA-treated rats, compared to the contralateral side. The recent report that ipsilateral spinal CB_2_ mRNA levels are decreased at later time-points in the mouse MIA model indicates that there are temporal changes in CB_2_ mRNA expression as OA progresses [24]. We report the first evidence for the expression of CB_2_ mRNA in the human spinal cord, and demonstrate a negative correlation with joint chondropathy. A positive correlation between the extent of chondropathy and pain has previously been reported [44], supporting the clinical utility of this approach. Chondropathy scores from patients in the current study (median per knee 112.4 (IQR 80 to 187)) were comparable to those reported in post-mortem cases (median 44 (IQR 18 to 87), and lower than those in most people presenting for joint replacement surgery (median 257 (IQR 228 to 283)) [32]. The negative association between CB_2_ mRNA levels and chondropathy **in human spinal cord** may reflect events associated with later stages of joint pathology, which is consistent with the reported observations at later stages of the MIA model [24]. Collectively clinical and pre-clinical evidence suggests that increased spinal CB_2_ mRNA expression early during the development of OA, as seen in the present study, may act to counter nociceptive signalling, whilst later reductions in spinal CB_2_ mRNA expression [24] may represent a failure of such homeostatic mechanisms and contribute to the progression of central sensitization and the manifestation of chronic OA pain. Changes in spinal CB_2_ receptor mRNA expression in OA suggest an important role of this target in regulating nociceptive processing in this disease, and our preclinical data indicate the therapeutic potential of CB_2_ agonists in relieving OA pain, at least at early stages of the disease. We also report a positive correlation of spinal GFAP mRNA expression and chondropathy score. As discussed earlier, astrocyte reactivity in the spinal cord is considered to be an important feature in the transition from acute to chronic pain mechanisms [9], [10]. The positive correlation between spinal GFAP expression and age may indicate, however, that changes in GFAP expression are driven by age, rather than joint chondropathy.

Immunohistochemistry studies presented herein provide new evidence for a significant increase in CB_2_ receptor protein expression in the ipsilateral spinal cord of MIA-treated rats. However control studies revealed that 10–20% of spinal CB_2_ positive cells present in the wild-type mouse were still present in spinal cord from CB_2_ knockout mouse, which suggests a lack of selectivity of the antibody or the incomplete knockout out of the CB_2_ receptor. Fluorescence labelling of the CB_2_ receptor with the novel probe NIR660-Mbc94 consolidated evidence for a significant increase in CB_2_ receptor protein expression in the ipsilateral spinal cord in this model of OA pain. NIR660-Mbc94 exhibited a moderate level of non-specific binding, as seen with other cannabinoid probes [45], [46]. Nevertheless, with appropriate experimental controls, this probe produces robust and reproducible binding in spinal cord sections and is an important new tool for the study of CB_2_ receptor protein in tissue *ex vivo*.

To further understand the mechanism(s) by which local activation of CB_2_ receptors attenuated nociceptive responses of spinal neurones in MIA-treated rats which had established chronic pain behaviour, but not in control rats, the cellular localisation of CB_2_ receptor protein was determined. The number of activated Iba-1 positive microglia and neurones (Neu-N) in the ipsilateral dorsal horn of the spinal cord which co-labelled the CB_2_ receptor antibody was significantly increased in MIA-treated rats, compared to the contralateral spinal cord and saline-treated rats. The contribution of glial cells to central sensitization is well known (see earlier), and the expression of CB_2_ receptors on (morphologically defined as activated) microglia is consistent with that reported for neuropathic pain models (see introduction). The expression of CB_2_ receptor protein by neurones (Neu-N) is in keeping with an earlier report [39] and the description of functional synaptic CB_2_ receptors in the CNS [47]. Our study demonstrates changes in spinal CB_2_ receptor expression by at least 2 different cell types, which provides multiple mechanisms by which agonists acting at this receptor can differentially regulate nociceptive processing in OA rats, compared to control rats. The inhibitory effects of the CB_2_ receptor agonist on pain behaviour were associated with a significant reduction in GFAP immunofluorescence in the spinal cord, indicative of a decrease in the level of reactive gliosis, which is associated with the maintenance of central sensitization in chronic pain states [27]. Furthermore, the ability of this treatment to attenuate the activation of spinal MMP-2 and MMP-9, which has been implicated in the activation of astrocytes [36], provides a novel mechanism by which activation of CB_2_ receptors dampens down central sensitization mechanisms, resulting in an attenuation of pain behaviour.

In conclusion, activation of CB_2_ receptors attenuated the development and maintenance of OA-induced pain behaviour. We provide electrophysiological evidence that acute activation of spinal CB_2_ receptors selectively attenuates spinal neuronal processing of noxious inputs in the established OA pain model. Mechanistic studies demonstrate the up-regulation of CB_2_ receptors in the spinal cord in this model of OA pain and suggest that CB_2_ receptor-mediated modulation of spinal neuro-immune responses contributes to the inhibitory effects of this target on OA pain responses. Our clinical and pre-clinical data support the further investigation of the potential of CB_2_ receptor agonists for the treatment of pain associated with OA, in particular at earlier stages of the disease.

## Supporting Information

File S1 **Supporting information.**
(DOCX)
