# Supplementary material for: Cannabinoid CB2 Receptors Regulate Central Sensitization and Pain Responses Associated with Osteoarthritis of the Knee Joint
Source: PLoS One. 2013 Nov 25;8(11):e80440. doi: 10.1371/journal.pone.0080440 (PMC3840025; doi:10.1371/journal.pone.0080440)
Supplement: File S1 — Supporting information. (DOCX) [file pone.0080440.s001.docx]

**Supplementary Information**

**CB_2_ knockout mice**

Knockouts were generated by homologous recombination, which results in deletion of 114 amino acid residues near the N terminus. Mice are identical to those that are commercially available through Jackson Laboratory (B6.129P2-Cnr2^tm1Dgen^/J), further information can be found on the Jackson Laboratory website (<http://jaxmice.jax.org/strain/005786.html>). Specifically the sequence encoding amino acids 26 – 140 of the CB2 receptor has been excised, although the coding sequence for amino acids 1 – 25 of the extreme N terminus remains.

**Primers and probes used for RT-PCR**

(Rat) CB_2_ forward primer - 5-TTGGCCGGAGCTGACTTC-3, (Rat) CB_2_ reverse primer – 5-CACACCGTGAAAGACATGGAA-3, (Rat) CB_2_ Taqman probe –5-CGTGATCTTTGCCTGCAACTTCGTCA-3.

(Rat) β-actin forward primer –5-AGGCCATGTACGTAGCCATCCA-3, (Rat) β-actin reverse primer – 5-TCTCCGGAGTCCATCACAATG-3, (Rat) β-actin Taqman probe – 5-TGTCCCTGTATGCCTCTGGTCGTACCAC -3.

(Human) CB_2_ forward primer - 5- AACACAACCCAAAGCCTTCTAGA -3, (Human) CB_2_ reverse primer – 5- GTCACCCAGCATTCCTCCAT -3, (Human) CB_2_ Taqman MBG probe 5- CTCAGTGGAATCTGAAGGG -3.

(Human) GFAP forward primer – 5- GGACCTGCTCAATGTCAAGCT-3, (Human) GFAP reverse primer – 5-TTTGCCCCCTCGAATCTG -3, (Human) GFAP Taqman probe – 5-ACCGGATCACCATTCCCGTGCA -3.

(Human) TRPV1 forward primer – 5- AGCACCTCACAGACAACGAGTTC-3, (Human) TRPV1 reverse primer – 5- TTTGCCGCGCGATCTC -3, TRPV1 Taqman probe (Human) 5- CTGCTGAAAGCCATGCTCAACCTGC-3.

(Human) COX2 forward primer – 5- GAATCATTCACCAGGCAAATTG -3, (Human) COX2 reverse primer – 5- TTTCTGTACTGCGGGTGGAAC -3, COX2 Taqman probe (Human) 5- TTCCTACCACCAGCAACCCTGCCA -3.

(Human) CB_1_ forward primer – 5- AGAAAGCAGGCGCCCTAAC -3, (Human) CB_1_ reverse primer – 5-TGATTAGGCTGAGCTCAAAATGACT -3, CB_1_ Taqman MGB probe (Human) 5- ATTGCCCCCTGTGGGT -3.

(Human) β-actin forward primer –5- CCTGGCACCCAGCACAAT-3, (Human) β-actin reverse primer – 5- GCCGATCCACACGGAGTACT -3, (Human) β-actin Taqman probe – 5- ATCAAGATCATTGCTCCTCCTGAGCGC -3.

**CB_2_ receptor probe synthesis**

The CB_2_ receptor targeted probe was developed by coupling a functional CB_2_ receptor ligand Mbc94 [1], with a near infrared (NIR) fluorescence dye NIR660 (SF4). The NIR dye exhibits negligible tissue autofluorescence in the NIR (650-900nm) region [2]. NIR660-Mbc94 has maximum absorption at 664 nm and emits at 786 nm (SF5). The large Stokes shift (122 nm) avoids the self-quenching and signal quantification issues of typical NIR fluorescent dyes, which have relatively small stokes shifts (usually less than 25 nm) [3].

**General:** Synthesis of NIR660 and Mbc94 were based on [1,4,5]. All chemicals were purchased from Sigma-Aldrich. Column chromatography was performed on the silica gel (standard grade, 60A, Sorbtech). ^1^H and ^13^C NMR spectra were recorded on the Brucker Avance III 400 MHz. MALDI-TOF mass spectra were recorded on a PerSeptive Voyager STR MS spectrometer. UV/Vis spectra were recorded on a Cary 100 Bio UV-Vis spectrophotometer, and fluorescence spectra were recorded on a Cary Eclipse fluorescence spectrophotometer.

**NIR660-Mbc94.** Dye NIR660 (21 mg, 22 μmol) was dissolved in freshly distilled DMF (5 ml) under argon and 2-(1*H*-benzotriazole-1-yl)-1,1,3,3-tetramethyl uronium hexafluorophosphate (HBTU) (11.4 mg, 30 μmol), *N*-Hydroxybenzotriazole (HOBt) (4 mg, 30 μmol), DIEA (5 μL, 30 μmol) and Mbc94 (15 mg, 25 μmol) were added and stirred at room temperature in dark for 18 hours. The solvent was removed by rotary evaporation, and the residue was purified by silica gel column chromatography (CH_2_Cl_2_ :MeOH, 15 :1 to 8 :1). The probe was further purified by precipitating over CH_2_Cl_2_/diethyl ether. Blue solid (8 mg, 24%) was obtained after drying in vacuo for 24 hours. ^1^H-NMR (MeOD-*d*4, 400 MHz): δ 8.16 (2H, d, *J* = 8.4 Hz), 7.90-7.86 (6H, m), 7.54 (2H, t, *J* = 8.4 Hz), 7.46 (2H, d, *J* = 9.2 Hz), 7.41-7.33 (5H, m), 7.30 (1H, d, *J* = 2 Hz), 7.17 (1H, dd, *J* = 2&8.4 Hz), 7.07 (2H, d, *J* = 8.4 Hz), 6.85 (1H, s), 5.91 (2H, d, *J* = 12.4 Hz), 5.49 (2H, s), 4.58 (1H, s), 4.13 (4H, t, *J* = 6.4 Hz), 4.08 (2H, s), 3.84 (2H, t, *J* = 6.4 Hz), 3.76 (1H, s), 3.11 (2H, t, *J* = 7.6 Hz), 2.89-2.84 (6H, m), 2.59 (4H, t, *J* = 6.4 Hz), 2.34 (3H, s), 2.21 (2H, t, *J* = 7.6 Hz), 2.02-1.96 (18H, m), 1.88-1.85 (4H, m), 1.79-1.78 (1H, m), 1.74-1.65 (5H, m), 1.54-1.43 (9H, m), 1.30-1.27 (6H, m), 1.15 (3H, s), 1.07 (3H, s), 0.85 (3H, s). MS (MALDI-TOF): calculated for [(M-Na^+^+2H^+^)^+^] 1495.4, found 1495.9.

**Filter cube for imaging NIR660-Mbc94**

A custom filter cube from Chroma Technology Corporation (Bellows falls, VT, USA) was used to image NIR660-Mbc94 binding in spinal cord sections. The excitation of this filter cube was 650/45X and the emission was 780IP (SF6).

**Confocal Microscopy**

Fresh frozen spinal cord sections (10μm) were cut on a cryostat and plated onto silane-prep slides (Sigma-aldrich, UK. S4651). Sections were equilibrated to room temperature for 1 hour and sections were then incubated with 1.5 mls of buffer A (50 mM Tris–HCl, 3 mM MgCl2, 0.2 mM EGTA, 100 mM NaCl) for 1 hour at room temperature. Buffer A was removed and 1.5 mls of 3μM NIR660-Mbc94 was added to the sections. 5 hours later NIR660-Mbc94 was removed and the sections were washed in buffer A. Sections were then either stained with Hoechst 33342 or cresyl violet.

Sections were incubated with 0.01 mg/ml Hoechst for 30 min, sections were then washed in 0.1 M PBS (phosphate buffered saline) then once in DDH_2_0. Sections were air dried, mounted in Polyvinyl alcohol mounting medium with NPG, antifading (Sigma-aldrich, UK. catalogue number: 10979) and coverslipped. Sections went on to be visualized and images captured with a confocal microscope.

Sections were incubated with cresyl violet (0.125 % made up in 500 ml DDH_2_0 + 200 μl glacial acetic acid) for 15 min. Sections were placed in DDH_2_0 for 3 min, air dried and then mounted in 200 μl of Polyvinyl alcohol mounting medium with NPG and coverslipped. Sections were visualized and images captured with a confocal microscope.

Images were acquired on a Leica SP2 TCS CLSM using a 63x 1.35 NA glycerol objective lens. Hoechst dye and NIR660-Mbc94 were excited using 405nm and 633nm lasers, respectively. Emission wavelengths detected were 410-460nm (for Hoechst) and 770-810nm (for NIR660-Mbc94). Cresyl violet staining of cell bodies was visualised using the 561 laser to acquire a TRANS image. For ease of visualisation an inverse greyscale LUT was applied.

**Validation of NIR660-Mbc94 binding to spinal CB_2_ receptors**

Competition binding curves demonstrated that 30-40% of NIR660-Mbc94 binding was blocked by the CB_2_ receptor agonist JWH133 and in separate experiments by the antagonist SR144528 (SF7A-D). In order to confirm the cellular distribution of NIR660-Mbc94 confocal microscopy revealed that NIR660-Mbc94 did not appear to localise to the nucleus of cells in the spinal cord (SF7E), but was present in cell bodies where it appeared more intense at their periphery (SF7F).

**Figures**


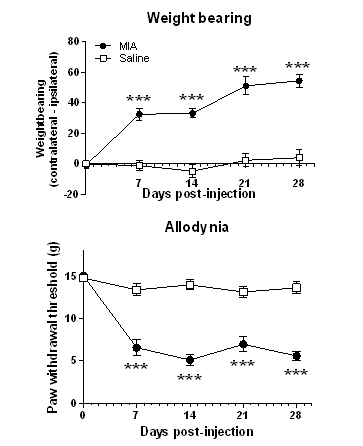


SF1: Intra-articular injection of MIA in the rat resulted in a significant decrease in ipsilateral weight bearing (top panel) and a decrease in ipsilateral hindpaw withdrawal thresholds (bottom panel), compared to saline-treated rats (n= 8-10 rats per group). Statistical analyses between groups: weight bearing (two way ANOVA with a Bonferroni post-hoc test); paw withdrawal thresholds (Kruskal Wallis test), ***p<0.001.

SF2: (A) A representative image of CB_2_ receptor antibody binding in the **rat** spinal cord (10 X magnification). (B) A representative image of CB_2_ receptor antibody binding in the spinal cord of a **wild type mouse** (10 X magnification). (C) A representative image of CB_2_ receptor antibody binding in the spinal cord of a **CB_2_ knockout mouse** (10 X magnification). Scale bar = 100 μM. N.B difference in scale bar length due to size differences between rat and mouse spinal cords.

SF3:

Localisation of CB_2_ receptor immunofluorescence with Iba-1 positive cells in spinal cord sections from wild-type (A) and CB_2_ receptor knockout (B) mice. Localisation of CB_2_ receptor immunofluorescence with NeuN positive cells in spinal cord sections from wild-type (C) and CB_2_ receptor knockout (D) mice. Scale bar = 10 μM.

SF4: Synthesis of CB_2_R probe **NIR660-Mbc94**

SF5: Absorption (solid curve) and emission spectra (dot curve) of NIR660-Mbc94 at concentration of 1×10^-6^ M in DMSO. λ_ex_=664 nm.


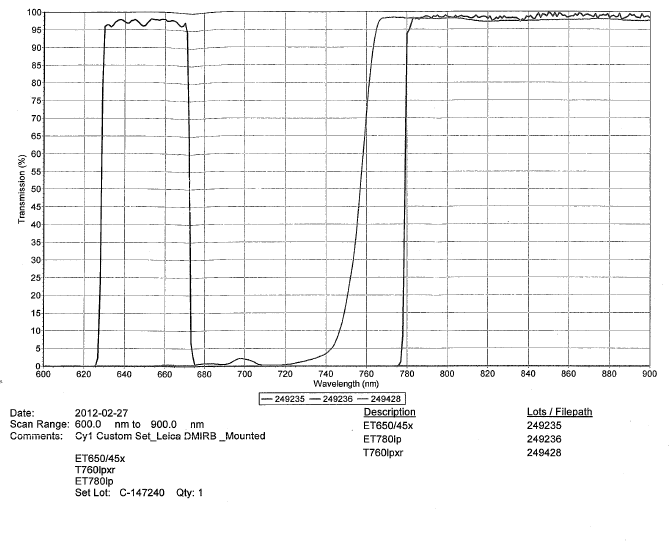
SF6: Technical data for custom filter cube used to visualize NIR660-Mbc94 binding.


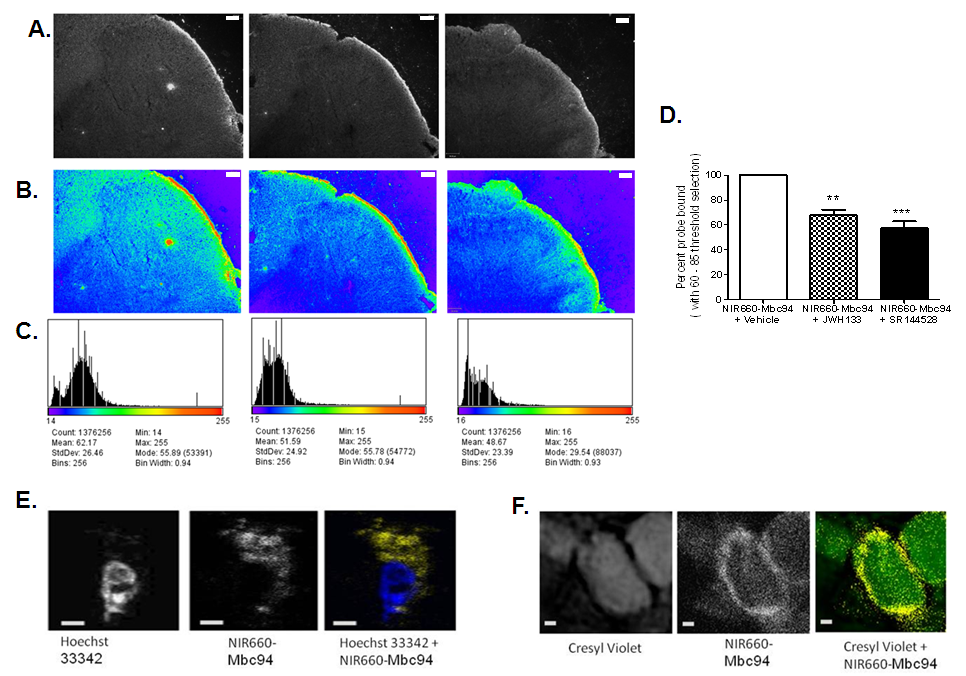


SF7: (A) Representative images of fresh frozen spinal cord sections treated with 3 µm NIR660-Mbc94, 3 µm NIR660-Mbc94 + 3 µm JWH133 or 3 µm NIR660-Mbc94 + 1 µm SR144528 (left to right). (B) ImageJ reassignment of sections from gray scale to rainbow plot for NIR660-Mbc94 localisation, low intensity pixels (14/16) are shown in blue purple and high intensity pixels (255) in red. (C) Histogram plots of sections in (B) demonstrating the differences in the pixel intensity between treatment groups (14,458 for CB_2_ probe + Vehicle, 3,281 for CB_2_ probe + JWH133 and 4,319 for CB_2_ probe + SR144528). (D) Quantification of NIR660-Mbc94 binding in the presence and absence of JWH133 or SR144528 (mean + SEM, 5-6 sections per condition). (E) Confocal images (63 x) of Hoechst 33342 (nuclear staining), NIR660-Mbc94 (CB_2_ probe staining) and merge (note the lack of co-distribution of Hoechst 33342 and NIR660-Mbc94 localisation), scale bar = 2 μm. (F) Confocal images (63 x) of cresyl violet (cell body staining), NIR660-Mbc94 and merge. Note the intense halo of NIR660-Mbc94 localisation around the edge of the cresyl violet stained cell body and the less intense punctuate NIR660-Mbc94 localisation that is seen nearer the centre of the cresyl violet stained cell body, scale bar = 2 μm.


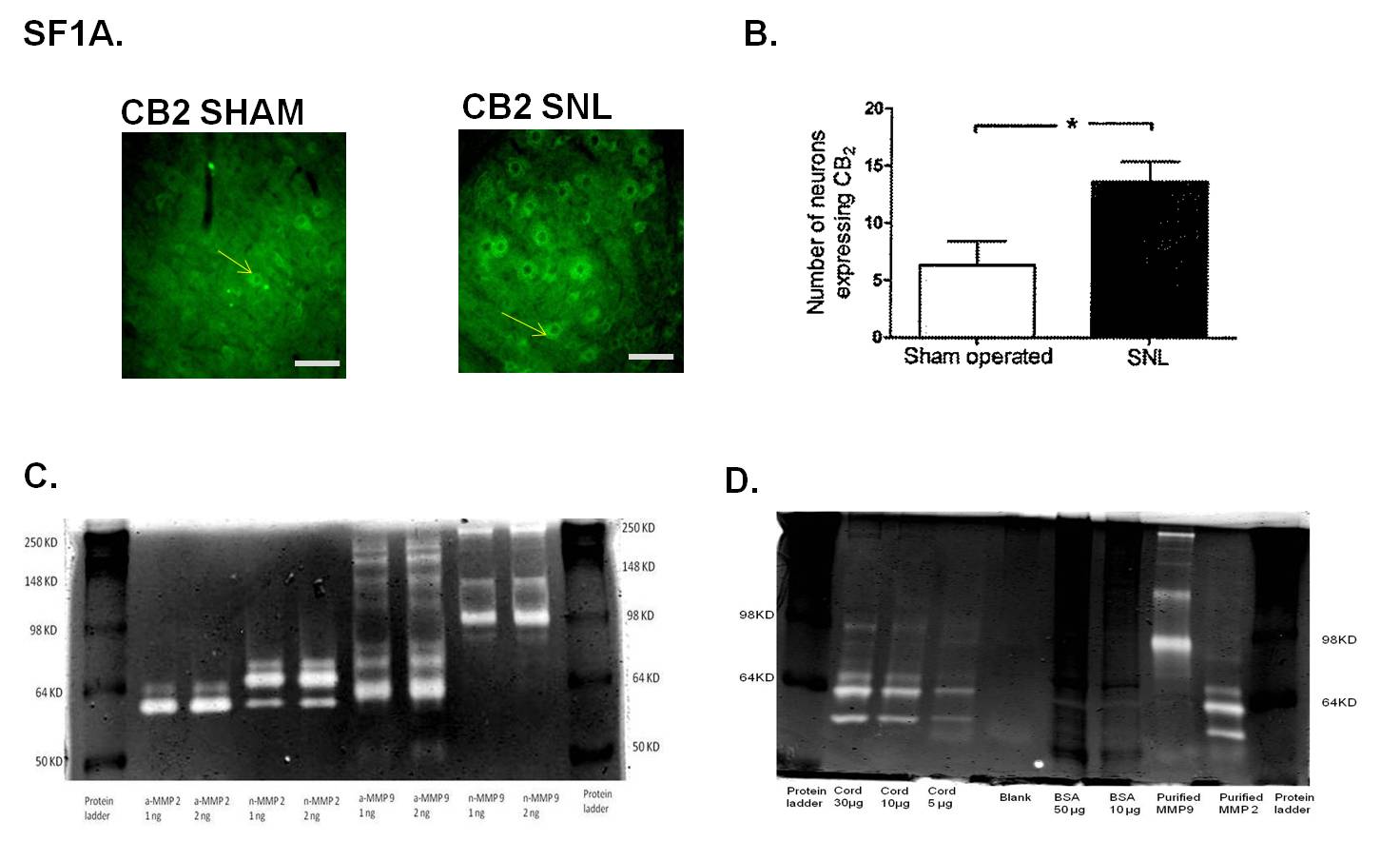

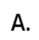

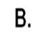


SF8A: Representative gel zymography blot showing bands for purified pro MMP 9, purified pro MMP2, artificially cleaved activated MMP9 and MMP2 and the protein ladder which serves as a negative control.

SF8B: Representative gel zymography blot of bands for spinal cord homogenate derived pro MMP 9, pro MMP2, active MMP2, two protein size marker bands and two BSA lanes, which serve as a negative control.

**Supplemental References**

1. Bai M, Sexton M, Stella N, & Bornhop DJ (2008) MBC94, a conjugable ligand for cannabinoid CB 2 receptor imaging. Bioconjug Chem 19(5):988-992.

2. Bai M & Bornhop DJ (2012) Recent advances in receptor-targeted fluorescent probes for in vivo cancer imaging. Curr Med Chem 19(28):4742-4758.

3. Peng X, et al. (2005) Heptamethine cyanine dyes with a large stokes shift and strong fluorescence: a paradigm for excited-state intramolecular charge transfer. J Am Chem Soc 127(12):4170-4171.

4. Masotti A, et al. (2008) A novel near-infrared indocyanine dye-polyethylenimine conjugate allows DNA delivery imaging in vivo. Bioconjug Chem 19(5):983-987.

5. Strekowski L, Mason CJ, Lee H, Gupta R, Sowell J, Patonay G. (2003) Synthesis of Water-Soluble Near-Infrared Cyanine Dyes Functionalized with [(Succinimido)oxy]carbonyl Group. J Heterocyclic Chem 40(5):913-916. (Unavailable through pubmed, please find here: http://onlinelibrary.wiley.com/doi/10.1002/jhet.5570400527/compoundindex)
